# Illegal and falsified medicines self-administrated in not approved post-cycle therapy after the cessation of anabolic-androgenic steroids – qualitative analysis

**DOI:** 10.3389/fchem.2025.1536858

**Published:** 2025-03-19

**Authors:** Agata Blazewicz, Magdalena Poplawska, Beata Daniszewska, Karolina Piorunska, Michal Karynski

**Affiliations:** Falsified Medicines and Medical Devices Department, National Medicines Institute, Warsaw, Poland

**Keywords:** anabolic androgenic steroids, AAS, post-cycle therapy, PCT, falsified medicines, cessation of AAS, SERMs, AIs

## Abstract

**Background:**

The term post-cycle therapy (PCT) often appears in bodybuilding forums in the context of anabolic-androgenic steroids (AAS) cessation. To reduce the negative impact of AAS on the hormonal system, unapproved PCT is used, which consist of medications that help restore hormonal balance. The most used medicinal products are selective estrogen receptor modulators (SERMs), aromatase inhibitors (AIs), and preparations containing human chorionic gonadotropin (hCG). These substances are prohibited in sports by the World Anti-Doping Agency.

**Methods:**

Between January 2020 and the end of August 2024, 601 samples seized by the police and prosecutor’s office from the illegal market, intended for use as performance-enhancing drugs (PEDs), were tested at the Polish Official Medicines Control Laboratory. Samples were analyzed using accredited methods, including liquid chromatography coupled with high-resolution hybrid mass spectrometry and X-ray powder diffraction, to estimate PCT drug prevalence among other PED samples. In total, 411 (68.4%) samples declaring to contain AAS, 63 (10.5%) declaring to contain substances used in PCT, and 127 (21.1%) other PEDs were tested.

**Results:**

Among the PCT drug samples, 33.3%, 25.4%, and 41.3% indicated the presence of SERMs (tamoxifen and clomiphene), AIs (anastrozole, letrozole, and exemestane), and other substances (hCG, cabergoline, and mesterolone), respectively according to the label. However, not all samples were consistent with the declarations. In 65.1% of the samples, the declared active pharmaceutical ingredients (APIs) were present, whereas in 34.9%, they were not. Furthermore, among the samples in which the declared API was found, 58.7% contained only the declared API, while 6.4% included an additional undeclared API. Conversely, among the samples without the declared API, 20.6% contained neither a declared API nor any API, while 14.3% had other undeclared APIs.

**Conclusion:**

We have shown that illicit drugs used in PCT may be substituted, adulterated, or contain no active ingredients. Our results indicate that in view of the high prevalence of illicit AAS use, the self-administration of unapproved PCT using illegal and falsified medicines is dangerous and can be considered a potential threat to consumer health.

## 1 Introduction

Anabolic-androgenic steroids (AAS) stimulate protein synthesis, leading to increased skeletal muscle mass and strength (anabolic effect). They are also responsible for the development of male secondary sexual characteristics (androgenic effect) (Hoffman and Ratamess, 2006). AAS have selected therapeutic uses, including male hypogonadism, anemia, and osteoporosis treatment. However, their anabolic effects have led to widespread non-medical use, especially among young adults engaged in athletic activities as performance-enhancing drugs (PEDs) (de Ronde and Smit, 2020; McCabea et al., 2007). Furthermore, AAS are banned according to the List of Prohibited Substances and Methods of the World Anti-Doping Agency (WADA) (World Anti-Doping Agency, 2024c).

Uncontrolled use of substances from the AAS group for non-medical purposes is associated with a high risk of adverse effects on the liver and the reproductive, musculoskeletal, cardiovascular, and central nervous systems. Exogenous AAS inhibit endogenous testosterone production, and discontinuation of their use is often associated with a prolonged decrease in testosterone secretion (Kanayama et al., 2015; Rasmussen et al., 2016; Shankara-Narayana et al., 2020). AAS-induced hypogonadism is a serious problem, and complete recovery is difficult in most cases (Vilar Neto et al., 2021). It can take months or years for the testosterone levels to return to normal after AAS cessation (Kanayama et al., 2015; Shankara-Narayana et al., 2020). Men who stop taking AAS may experience decreased libido, erectile dysfunction, fatigue, and depression symptoms (Kanayama et al., 2015; Rasmussen et al., 2016). Since no evidence-based recommendations for safe AAS withdrawal management currently exist, some users self-administer non-approved post-cycle therapy (PCT) to stimulate endogenous testicular function (Griffiths et al., 2017; Grant et al., 2023a).

The substances used in PCT are administered in the treatment of male hypogonadism unrelated to androgen abuse (Grant et al., 2024). However, PCT is recommended in bodybuilding forums for men who have discontinued testosterone or other androgenic hormones. Moreover, the PCT protocol differs depending on the individual group. Recreational AAS users (men without hormone therapy indications) predominantly start with optimal testosterone levels, aiming to quickly improve their physique. However, they experience a decrease in endogenous testosterone production owing to AAS use, which manifests, among others, as erectile dysfunction and spermatogenesis suppression, resulting in testicular atrophy, and infertility. PCT is believed to prevent testicular atrophy and inhibits feminization processes, such as gynecomastia. PCT can become even more important when high AAS doses are used or when multiple AAS are administered simultaneously.

To restore endogenous testosterone, mainly aromatase inhibitors (AIs), selective estrogen receptor modulators (SERMs), and human chorionic gonadotropin (hCG) are used (Karavolos et al., 2015; Bonnecaze et al., 2021). AIs and SERMs are relatively new doping agents, similar to selective androgen receptor modulators (SARMs) (Hackney, 2018).

In the 2024 WADA Prohibited List (World Anti-Doping Agency, 2024c) Section 4: “hormone and metabolic modulators,” AIs (Subsection S4.1) and anti-estrogenic substances, including anti-estrogens and SERMs (Subsection S4.2), are prohibited at all times (in- and out-of-competition). In Subsection S2.2.1, chorionic gonadotropin is listed as a testosterone-stimulating peptide in males.

Aromatase is an enzyme that converts testosterone to estradiol (the most potent estrogen form) and androstenedione to estrone (Kang et al., 2017). As aromatase mediates estrogen production, its inhibition with chemical molecules is considered an effective treatment for estrogen receptor (ER)-positive breast cancer. Moreover, AIs are used off-label to reduce circulating estradiol levels, thereby inhibiting the negative feedback of AAS use, limiting excessive testosterone release. Therefore, AIs play a role in minimizing the side effects associated with elevated estradiol levels in men, including gynecomastia (Basaria, 2010). AIs can be divided into two groups based on their chemical structure: steroidal (analogs of androstenedione, exemestane, formestane, and testolactone), which provide irreversible aromatase inhibition, and non-steroidal (anastrozole and letrozole), which provide reversible inhibition (Ahmad, 2015). The main AIs currently used include the new-generation letrozole, anastrozole, and exemestane, which can be administered orally.

The anti-estrogenic compounds mentioned in the WADA list can be divided into two groups: SERMs (bazedoxifene, clomiphene, cyclofenil, ospemifene, raloxifene, tamoxifen, and toremifene) and selective estrogen receptor degraders (SERDs), of which fulvestrant is a representative. Fulvestrant is the first SERD approved for clinical use to treat advanced and metastatic breast cancer. SERMs are non-steroidal estradiol analogs with mixed agonist-antagonist effects on ERs and are used in osteoporosis and breast cancer treatment (Jordan, 2004). Tamoxifen was the first SERM used to treat ER-positive breast cancer (Hackney, 2018). Clomiphene and cyclofenil are also older SERMs (although perhaps less selective than tamoxifen) (Heuberger and Cohen, 2019). In approved medicines, clomiphene is a mixture of two isomers: cis (Z-isomer or zuclomiphene) and trans (E-isomer or enclomiphene). Enclomiphene is anti-estrogenic, whereas zuclomiphene manifests moderate estrogenic and anti-estrogenic effects (Girase et al., 2023). Raloxifene is a new SERM developed for osteoporosis that appears to prevent breast cancer (Lewis and Jordan, 2005). Similar to AIs, SERMs are used off-label to alleviate the side effects of excess estradiol. The main antiestrogenic substances can be administered orally.

In addition, other medicines are recommended on bodybuilding forums during PCT. hCG is used by bodybuilders and athletes at the end of an AAS cycle to prevent muscle tissue breakdown. hCG directly stimulates Leydig cells to produce and release intratesticular testosterone by binding to the luteinizing hormone/choriogonadotropin receptor (Rizzuti et al., 2023). Moreover, hCG maintains spermatogenesis during gonadotropin suppression (Bond et al., 2022). However, according to bodybuilder forums, hCG is also important in restoring testicles to their normal size, as often, after a period of AAS use, an athlete’s testicles can shrink. Chorionic gonadotropin is a heterodimeric glycoprotein composed of two non-covalently bound subunits. The α subunit of hCG is structurally identical to the α subunits of gonadotropins secreted by the pituitary gland. The β subunit differs in its amino acid sequence from other gonadotropins and is hCG-specific. However, posttranslational modifications with eight potential glycosylation sites in the hCG structure result in a wide range of hCG glycoforms.

When administering 19-nortestosterone derivatives with significant progestogenic activity (nandrolone and trenbolone), cabergoline is recommended. Cabergoline is an ergot alkaloid derivative used to treat hyperprolactinemia, prolactinoma, and Parkinson’s disease. It effectively inhibits prolactin secretion and is therefore used by men during PCT to prevent gynecomastia. Bromocriptine, an ergot alkaloid derivative used to treat hyperprolactinemia, has a structure and activity similar to cabergoline. Mesterolone is used not only to increase androgen levels in athletes as an AAS but also for its supposed intrinsic aromatase enzyme antagonist properties as an anti-estrogen (Llewellyn, 2017). To the best of our knowledge, there is only one available source (outside of sports blogs and forums) that provides information on aromatase inhibition by mesterolone (Llewellyn, 2017).

Medicines used in PTC may cause serious side effects, including blood clots, stroke, and endometrial cancer in women taking SERMs (Hackney, 2018). The main adverse events reported by male AAS users to SERMs (clomiphene and tamoxifen) were decreased libido and erectile dysfunction (24%), acne (21%), fatigue (19%), mood disorders (13%), visual disturbances (5%), arthralgia (4%), insomnia (4%), and hot flushes (2%). Two men reported serious adverse drug reactions (hepatic cytolysis requiring hospitalization and abdominal pain requiring a visit to the emergency department) (Rochoy et al., 2022). In another study based on website data, AAS users reported tamoxifen side effects such as blurred vision, dizziness, headaches, and reduced libido (Karavolos et al., 2015). The main adverse events reported on AIs (anastrozole, letrozole, and exemestane) were decreased libido (27%), arthralgia (24%), mood disorders (12.5%), asthenia (10%), headaches (6%), anxiety (4.5%), and palpitations (3%). Three men reported serious adverse drug reactions requiring an emergency department visit (gross hematuria, palpitations, and urticaria) (Rochoy et al., 2022). According to other study AAS users reported arthralgia as an AI side effect (Karavolos et al., 2015). Furthermore, hCG can cause or aggravate gynecomastia (Rahnema et al., 2014). Adverse effects of chronic dopamine agonist (cabergoline) use include headaches, orthostatic hypotension, nausea, and sometimes cardiac valvular disease (Bonnecaze et al., 2021).

Owing to restrictions on the purchase of AAS and PCT products, an international black market for such preparations has developed. Unfortunately, because AAS cycles and PCTs are carried out outside the supervision of the doctor, most often using illegally traded products, they can be considered a potential threat to consumer health. The availability of illegally sold medicines has become a challenge in recent decades. For many years, falsified medicines have been traded worldwide; however, this has become increasingly easier owing to the increased availability of medicines and open channels of trade, including online sales. In addition to drug trafficking, pharmaceutical crime is of interest to organized criminal groups, and many factors contribute to the falsification of medicines. It is generally stated that over 50% of medical products sold through websites other than established online pharmacies can be classified as falsified medical products (Deconinck et al., 2021). The World Health Organization distinguishes between three categories of poor-quality medical products: substandard, unregistered/unlicensed, and falsified (World Health Organization, 2018).

The necessity of identifying unknown and undeclared substances in illegal and falsified products requires the constant development of techniques used for analytical research in forensic or control laboratories to perform analyses for public authority needs. Mass spectrometry (MS) has long been used for the identification and determination of the molecular structure of organic compounds (Khalikova et al., 2024). Different analyzers used in high resolution MS, including time of flight (TOF), and Orbitrap enable the determination of the elemental composition (assignment of the most probable sum formula) of a compound by measuring the exact mass and comparing the isotopic profile of the obtained spectrum with the theoretical profile. In many cases, the analysis of fragmentation spectra allows for the determination of the structural formula of the compound or elements of its structure. In the identification of unknown compounds, the use of high-resolution hybrid quadrupole (Q)-TOF, additionally coupled with high performance liquid chromatography (LC-QTOF-MS/MS) is irreplaceable (Johansson et al., 2014; Rebiere et al., 2017). However, to confirm the structure of the unknown compounds, especially those for which reference materials are not available, nuclear magnetic resonance (NMR) spectrometry is recommended (Johansson et al., 2014; Rebiere et al., 2017; dos Santos Ribeiro et al., 2021). X-ray powder diffraction (XRPD) enables the identification of crystalline substances, including both active pharmaceutical ingredients and excipients, that can be lost during dissolution and filtration when preparing samples for other analytical techniques. It also allows the determination of the form in which a compound is present, whether as a salt or a co-crystal. The Polish Official Medicines Control Laboratory (OMCL), upon request by police, customs, or other enforcement groups, regularly tests AAS and other PEDs submitted to the laboratory using accredited methods such as LC-QTOF-MS/MS and XRPD.

Although a growing body of literature on PCT and user characteristics associated with PCT use exists (Griffiths et al., 2017; Bonnecaze et al., 2021; Grant et al., 2023a), data on the quality of medications used within PCT are limited, in contrast to AAS quality (Graham et al., 2009; Frude et al., 2020; Magnolini et al., 2022). This study aimed to estimate the prevalence of medicines used for non-approved PCT among other PED samples and to identify declared and undeclared substances in illicit samples to check whether they were in accordance with the declaration. In this report, we describe the results of these studies to raise awareness that illegal and falsified products that pose a risk to consumer health exist in the European market.

## 2 Materials and methods

### 2.1 Scope

Samples declared as containing substances intended to enhance sports performance, such as AAS, other PEDs, or substances used after the AAS cycle to mitigate side effects, were included. Active ingredient identification was conducted for all samples, however only the results for the PCT samples are presented in this study. Substances of abuse banned by the WADA List (World Anti-Doping Agency, 2024c), such as stimulants from Section S6, narcotics from S7, and cannabinoids from S8, were excluded. In addition, phosphodiesterase-5 inhibitors (PDE-5i), popular among AAS users, were excluded, although they were thought to increase libido after AAS cessation.

### 2.2 Samples and timeframe

All samples were obtained between January 2020 and the end of August 2024 from the illegal supply chain seized by the police, prosecutor offices, customs, or other enforcement groups. Products were collected from illicit distribution sites for medicinal products, online stores, individual mail shipments, and prisons.

### 2.3 Chemicals

Methanol and acetonitrile, both of purity suitable for LC–MS, were purchased from Merck Millipore (LiChrosolv; Darmstadt, Germany) or Honeywell (Seelze, Germany), formic acid and ammonium bicarbonate for LC-MS from Honeywell, dithiothreitol (DTT) from Pierce Biotechnology (Rockford, IL, United States), iodoacetamide from Sigma-Aldrich (St. Louis, MO, United States), and trypsin from Roche Diagnostics (Mannheim, Germany). Ultrapure water (18.2 MΩ cm resistivity, Arium Comfort H2O-I-1-UV-T from Sartorius, Goettingen, Germany) or LC-MS grade water (LiChrosolv, Merck KGaA) was used throughout.

Reference standards: anastrozole, cabergoline, clomiphene citrate, letrozole, mesterolone, and raloxifene hydrochloride (EDQM, Strasbourg, France); tamoxifen citrate (Sigma Aldrich, Saint Louis, MO, United States); exemestane (USP, Rockville, MD, United States); EDTA (Chempur, Piekary Śląskie, Poland); WHO International Standard for hCG; and medicinal product Pregnyl (N.V. Organon, Netherlands).

### 2.4 Sample preparation

For LC–QTOF-MS/MS analysis, 1 mg of powder or homogenized tablet was dissolved or extracted using 5 mL of a 1:1:1 (v/v/v) mixture of water/methanol/acetonitrile, assisted by ultrasonication for 10 min, and afterwards filtered by a Whatman 0.2 μm pore size polytetrafluoroethylene (PTFE) filter medium (GE Healthcare, Chicago, IL, United States). The filtrate was diluted to suitable concentrations when necessary. Samples suspected to contain hCG were dissolved in water and cleaned by centrifugation.

For XRPD analysis, the powder samples or homogenized tablets were placed in circular plastic holders (1.5 mm deep, 25 mm in diameter) and leveled with a microscope slide. These holders were designed to minimize background interference.

### 2.5 Apparatus and parameters

#### 2.5.1 LC-QTOF-MS/MS

The parameters of LC-QTOF-MS/MS methods depended on the equipment on which the analyses were carried out. Two screening methods and hCG detection methods (intact and tryptically digested) were applied. Chromatographic and mass spectrometric parameters are summarized in the Table 1. In each method solvents A and B comprising of water-acetonitrile-formic acid (90:10:0.1, v/v/v) and methanol-acetonitrile-formic acid (90:10:0.1, v/v/v), respectively were used.

**TABLE 1:** Summarized chromatographic and mass spectrometric parameters of analytical methods applied in the study.

Methods	Screening method used in 2020–2023	Screening method used in 2024	hCG detection (tryptically digested)	hCG detection (intact)
LC parameters				
Column	Hypersil GOLD C18 analytical column 100 × 2.1 mm, 3 μm particle size with a guard column (both from Thermo Fisher Scientific)	Shim-pack Velox C18 150 × 2.1 mm, 1.8 μm particle size (Shimadzu)	Shim-pack Velox C18 150 × 2.1 mm, 1.8 μm particle size (Shimadzu)	Supelco BIOshell A400 Protein C4 column 100 × 2.1 mm, 3.4 μm particle size (Merck KGaA)
Gradient program	0–2 min—10%B 2–7 min—10–90%B 7–10 min—90%B 10–12 min—90–10%B 12–14 min—10%B	0–2 min—0%B 2–9 min—0–90%B 9–11 min—90%B 11–12 min—90–0%B 12–15 min—0%B	0–2 min—0%B 2–60 min—0–60%B 60–65 min—100%B 65–68 min—0%B	0–2 min—0%B 2–50 min—0–32%B 50–54 min—32%B 54–55 min—32–0%B 55–60 min—0%B
Oven temperature	40°C	40°C	40°C	70°C
Flow rate	0.15 mL min^−1^	0.35 mL min^−1^	0.20 mL min^−1^	0.30 mL min^−1^
PDA	200–320 nm	190–400 nm	190–400 nm	200–320 nm
MS parameters				
ESI source	drying gas flow rate: 8.0 L min^−1^ dry heater: 180°C capillary voltage: 4500 V (pos)/3200 V (neg) end plate offset: −500 V	drying gas flow rate: 10.0 L min^−1^ nebulizing gas flow rate: 3.0 L min^−1^ heating gas flow rate: 10.0 L min^−1^ interface temperature: 300°C DL temperature: 250°C heat block: 400°C interface voltage: 4,000 V (pos)/3,500 V (neg)	drying gas flow rate: 10.0 L min^−1^ nebulizing gas flow rate: 3.0 L min^−1^ heating gas flow rate: 10.0 L min^−1^ interface temperature: 300°C DL temperature: 250°C heat block: 400°C interface voltage: 4,000 V (pos)	drying gas flow rate: 9.0 L min^−1^ nebulizer: 1.6 Ba dry heater: 200°C capillary voltage: 4500 V (pos) end plate offset: −500 V
MS mode	full scan mode (m/z 50–1,500) Auto-MS/MS with five Precursor Ions (intensity order)	full scan mode (m/z 50–1,500) DDA with six MS/MS events (intensity order)	full scan mode (m/z 200–1,500) DDA with six MS/MS events (intensity order) detected in a range of m/z 100–2,200	full scan mode (m/z 900–3,000) without MS/MS events
CE	Linear gradient: m/z 200–20eV m/z 400–25 eV m/z 800–35 eV	35V ± 20V	20V ± 5V	-

pos – positive ionization mode; neg – negative ionization mode; MeOH, methanol; ACN, acetonitrile; CE, collision energy; DDA, Data Dependent Analysis algorithm; DL, desolvation line.

##### 2.5.1.1 Small molecules analysis

Compounds in the samples analyzed between 2020 and 2023 were identified using a high-performance liquid chromatograph (Ultimate 3,000 system from Dionex, Thermo Fisher Scientific, Waltham, MA, United States) coupled with a high-resolution micrOTOF-QII hybrid mass spectrometer (Bruker Daltonik, Bremen, Germany). The screening method described in our previous study (Poplawska et al., 2020) was applied with the LC and MS parameters described in the Table 1. TOF analyzer was calibrated prior to each sample using a solution of sodium formate.

Compounds in the samples analyzed in 2024 were identified using a liquid chromatograph with quadrupole TOF mass spectrometer (LCMS9050-Q-TOF, Shimadzu Corporation, Kyoto, Japan). Chromatographic and mass spectrometric parameters of this screening method are summarized in the Table 1. A calibration segment with a sodium formate solution was included in each sample run to ensure high mass accuracy.

Both targeted and non-targeted data analysis was applied. In the first step, a targeted screening was conducted using an in-house database to find known compounds potentially present in the sample. This was followed by a thorough data analysis for unidentified components of the sample. Retention times, full scans, and MS/MS spectra were compared with those obtained from the analysis of reference standards or suitable databases such as PubChem or MassBank, which are public repositories of mass spectra.

##### 2.5.1.2 Large molecules analysis

Regarding samples suspected of containing large peptides or proteins, the HPLC column was changed to a Supelco BIOshell A400 Protein C4 column (10 cm × 2.1 mm; 3.4 μm particle size; Merck KGaA, Darmstadt, Germany), MS settings of the screening method used in 2020–2023 were switched to high-mass detection (m/z 700–3,000) and the ESI-L LCMS Tuning Solution (Agilent Technologies) was used for calibration, whereas, in the screening method used in 2024 the data acquisition range was changed to m/z 100–3,000, and a sodium iodide solution was used for MS calibration.

##### 2.5.1.3 Intact hCG analysis

Samples purported to contain hCG were subjected to direct LC-QTOF-MS/MS analysis without prior pretreatment. Analyses were performed using an Ultimate 3000 HPLC system (Dionex) coupled with a micrOTOF-QII system. The settings described in the Table 1 were adjusted according to the conditions described by other researchers (Camperi et al., 2018).

##### 2.5.1.4 hCG tryptic digestion

One mg of the sample was dissolved in 200 µL of 50 mM ammonium carbonate. Then, 5 µL of 200 mM DTT was added and incubated for 40 min at 60°C; subsequently, 5 µL of 1 M iodoacetamide was added and incubated for 1 h, at room temperature and protected from light. At the end of this step, 20 µL of 200 mM DTT was added before digestion with 50 µL trypsin overnight, at 37°C. The enzymatic reaction was stopped by adding 5 µL of formic acid, and the sample was filtered by 0.2 μm PTFE filters. LCMS9050-Q-TOF analyses were conducted using similar parameters as in screening method described above, except for the gradient program. Details are provided in the Table 1. Raw data were directly loaded into Peaks Studio 11 software (Bioinformatics Solutions Inc., Canada), which enables peptide and protein identification, *de novo* peptide sequencing, and database searches. Carbamidomethylation was set as a fixed modification, whereas methionine oxidation, asparagine and glutamine deamination, and glycosylation options were set as variable modifications, according to published data (Lebede et al., 2021). Trypsin was defined as the cleavage enzyme; a maximum of two missed cleavages and three variable posttranslational modifications per peptide were allowed. The precursor and fragment mass error tolerances were set to 10 ppm and 0.1 Da, respectively.

The intact hCG analysis revealed several hCGα glycoforms; however, hCGβ was not detected owing to a poor ionization in the ESI source and high heterogeneity of hCGβ isoforms, consistent with previously published studies (Camperi et al., 2018). Nevertheless, various studies reported that an extensive pre-purification (Toll et al., 2006) or specific solid-phase extraction (Goumenou et al., 2025) yielded a mass spectrum of the intact subunit β. To resolve the hCGβ detection problem, a tryptic digestion of the sample, monitoring of the peptide or peptides unique for hCGβ, and using them as hCG markers were applied in several studies (Gam et al., 2003; Lund et al., 2012). Here, we performed tryptic digestion of potential hCG-containing samples and applied the PEAK Studio software with a database search to identify the protein. Even without introducing a post-translation glycosylation option, the hCGβ subunit could be detected in the Pregnyl sample (reference sample) with a 67% sequence coverage. When potential glycosylation was allowed in the settings, the sequence was covered in 98%.

#### 2.5.2 XRPD

All XRPD experiments were performed using a Bruker AXS D8 Advanced diffractometer equipped with a copper X-ray tube and a Våntec position-sensitive detector. The instrument operated at 40 kV and 40 mA, generating monochromatic CuKα radiation. Data collection was conducted in θ-θ mode following the Bragg‒Brentano method, within the 2 θ range of 4.8°–60°, using a step size of 0.011° and a time per step of 424 s. The collected diffraction patterns were subjected to background correction and standard smoothing, and the resulting data were analyzed using DIFFRAC.EVA V4.1 software, comparing them to both the PDF-2 commercial database and an in-house reference library.

### 2.6 API identification

Qualitative composition tests were conducted using LC-QTOF-MS/MS and XRPD methods to identify components, especially those that were not declared. Retention times, full scans, and MS/MS spectra (for LC-QTOF-MS/MS) or diffractograms (for XRPD) were compared with those obtained from the analysis of reference standards or suitable databases such as PubChem, MassBank, or PDF-2, which are public repositories of mass spectra or commercial databases of diffraction patterns, respectively.

Samples were tested for the presence of AIs (2-androstenol (5ɑ-androst-2-en-17-ol), 2-androstenone (5ɑ-androst-2-en-17-one), 3-androstenol (5ɑ-androst-3-en-17-ol), 3-androstenone (5ɑ-androst-3-en-17-one), 4-androstene-3,6,17 trione (6-oxo), aminoglutethimide, anastrozole, androsta-1,4,6-triene-3,17-dione (androstatrienedione), androsta-3,5-diene-7,17-dione (arimistane), exemestane, formestane, letrozole, and testolactone), and anti-estrogenic substance (bazedoxifene, clomiphene, cyclofenil, fulvestrant, ospemifene, raloxifene, tamoxifen, and toremifene) prohibited by WADA.

In addition, the samples were screened for other AIs and anti-estrogenic substances presence because the WADA list is open and not limited to the above-mentioned substances.

## 3 Results

### 3.1 Classification of samples by declaration of the label

**FIGURE 1:**
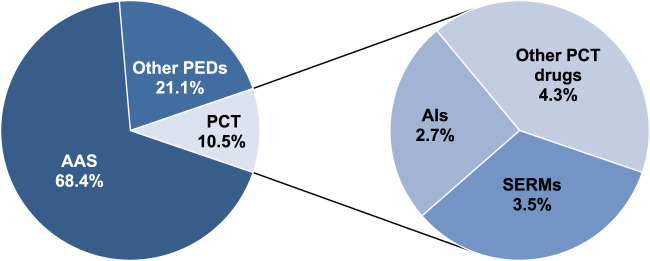
Distribution of samples (2020–2024) based on the substance declaration on the packaging.

A total of 601 samples declared as containing PEDs were obtained. Our classification of the various PED substances, based on the packaging declaration, resulted in three main groups: AAS, medicines used in PCT, and other PEDs. Most of the samples (n = 411, 68.4%) indicated AAS presence; however, 63 (10.5%) were intended for PCT, and 127 (21.1%) were declared as containing other PEDs (Figure 1.). These substances are briefly described as follows:

#### 3.1.1 AAS

Reporting the identified AAS results was not the subject of this study, except for mesterolone, which bodybuilders use as a PCT drug. Therefore, mesterolone was included as a PCT substance. Other AAS were only counted as AAS according to the packaging declaration for this project.

#### 3.1.2 Other PEDs

This group included other substances prohibited by WADA, such as non-approved substances (BPC-157) from Section S0, other anabolic agents (clenbuterol and SARMs) from Subsection S0, S1.2, peptide hormones and their releasing factors (AOD-9604, hGH 176-191, CJC-1295, ibutamoren, ipamorelin, and growth hormone-releasing peptide) from S2.2, growth factors and growth factor modulators (thymosin-ß4 and its derivative TB-500) from S2.3, beta-2 agonists (salbutamol and indacaterol) from S3, metabolic modulators (meldonium, GW1516, SR9009, SR9011, and insulins) from Section S4.4, and thyroid hormones.

#### 3.1.3 PCT drugs

This group comprises AIs, SERMs, and other PCT substances, such as hCG, cabergoline, and mesterolone. The results of the analyses of these samples are presented in the following sections.

PCT drugs were declared in 63 samples (10.5%). Of the 63 samples, SERMs were declared in 21 (3.5%), AIs in 16 (2.7%), and other PCT substances in 26 (4.3%) (Figure 1).

### 3.2 Annual comparison of PEDs samples (2020–2024)

**FIGURE 2:**
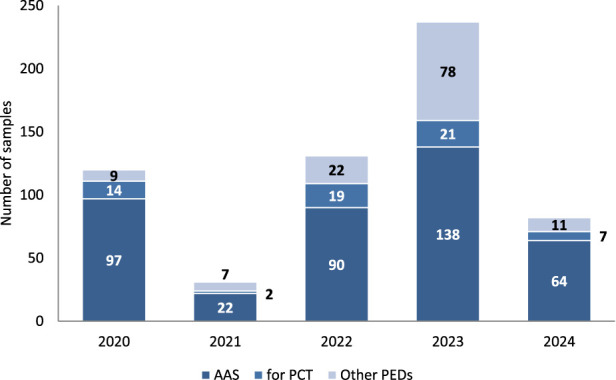
Number of samples from different PED groups submitted at Polish OMCL each year. All, n = 601; 2020, n = 120; 2021, n = 31; 2022, n = 131; 2023, n = 237, 2024, n = 82. *Data for 2024 is only through 31 August 2024.

The numbers of the three types of samples provided for each year are summarized in Figure 2.

### 3.3 Dosage forms of the PCT samples

**FIGURE 3:**
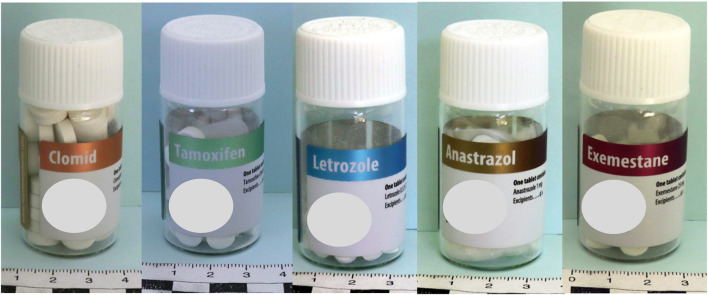
Examples of PCT samples in tablet forms.

Almost all the PCT samples had a pharmaceutical product appearance. However, some products had strange battery-like appearances with the writing “get more power” and signs “+” and “-” on both sides. Most of the tested samples (74.6%, n = 47) were in tablet form intended for oral administration (Figure 3), while 25.4% were presented as powders for injections (n = 16).

### 3.4 Packaging and product authentication of the PCT samples

Most packaging was without leaflets (71%; n = 45); however, 18 samples (29%) had leaflets, including 13 in Greek, 2 in Turkish, 1 in English, and 2 in Polish. Some of the external product packaging contained anti-counterfeit markings, located on stickers under the scratch-off label with “the anti-counterfeit number” and instructions: “*Scratch coating on the anti-counterfeiting label to get the anti-counterfeit number. Visit our website to check its authenticity,”* “*Before use, please authenticate your product at our website (link), authentication code, scratch here”* or *“Please use the Authentication system to verify the products you use are real, each time. This is the only way to ensure you are not using a fake product and the product is safe to use.”* After verification on the manufacturer’s website, the products are confirmed as authentic with messages such as “*This serial number exists. You got the real product.”* Many evidential products did not meet the labeling requirements for original medicinal products (including no batch number, list of excipients, full manufacturer name, information leaflets, and permit number on the packaging). Moreover, mistakes were mainly in the names of active pharmaceutical ingredients (APIs), such as *exemastene* instead of exemestane, *anastrazole* instead of anastrozole, and others.

### 3.5 AI identification

Among the 63 samples, three AI APIs (anastrozole, letrozole, and exemestane) were identified using LC-QTOF-MS/MS (Table 2). These substances are used in small doses (mostly 1 mg anastrozole, 2.5 mg letrozole, and 12.5 mg exemestane); therefore, the concentration was too low for XRPD.

**TABLE 2:** List of seized products and identified active substance(s) in the study.

Item	Product labeling—declared API	Declared API identified	Undeclared API(s) identified
Products with aromatase inhibitors			
1	Letrozole	yes	exemestane, yohimbine
2	Letrozole	yes	no
3-4	Anastrozole	yes	no
5	Anastrozole	yes	tamoxifen, oxandrolone, ligandrol, cardarine
6	Anastrozole	yes	tamoxifen
7	Anastrozole	no	letrozole
8	Anastrozole	no	letrozole, stanozolol
9	Anastrozole	no	letrozole, ostarine
10-16	Exemestane	yes	no
Products with anti-estrogenic substances			
17-21	Tamoxifen	yes	no
22	Tamoxifen	no	EDTA-Na
23	Tamoxifen	no	no
24-30	Clomiphene	yes	no
31-34	Clomiphene	no	no
35	Clomiphene	no	methyltestosterone, stanozolol
36	Clomiphene	no	tadalafil, sildenafil
37	Clomiphene	no	methandienone, sildenafil
Products with other substances used in PCT			
38-40	Cabergoline	yes	no
41	Cabergoline	yes	stanozolol
42	Cabergoline	no	no
43	Cabergoline	no	sildenafil
44-45	Mesterolone	yes	no
46	Mesterolone	no	stanozolol, sildenafil
47	Mesterolone	no	no
48-57	hCG	yes	no
58-63	hCG	no	no
Products with undeclared API for PCT			
64	Chlorodehydromethyltestosterone	yes	exemestane, oxandrolone
65	Chlorodehydromethyltestosterone	yes	tamoxifen
66	Oxandrolone	no	tamoxifen
67	Oxandrolone	yes	tamoxifen, chlorodehydromethylotestosterone
68	Oxandrolone	yes	tamoxifen, stanozolol
69	Liotyronine	no	tamoxifen
70	BPC-157	yes	raloxifene, ibutamoren, SR9009
71-72	Chlorodehydromethyltestosterone	yes	raloxifene
73	Dapoxetine	yes	clomiphene
74-75	Tadalafil	yes	clomiphene, dapoxetine
76	Sildenafil	yes	clomiphene
77	Cardarine	yes	clomiphene

#### 3.5.1 Letrozole

**FIGURE 4:**
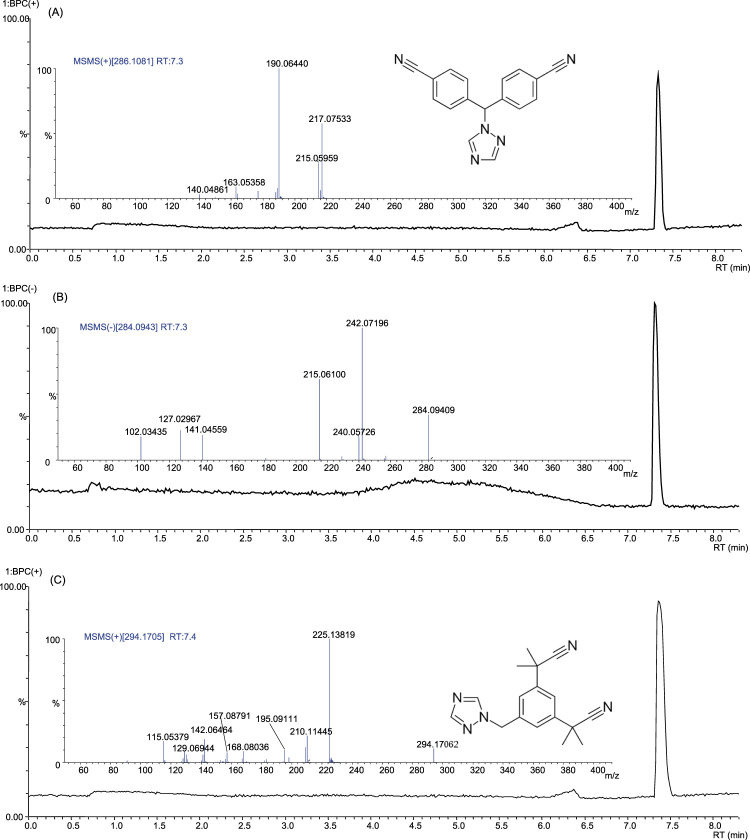
Base peak chromatograms and product ion spectra of letrozole in positive ion mode **(A)**, negative ion mode **(B)** and of anastrozole in positive ion mode **(C)**.

**FIGURE 5:**
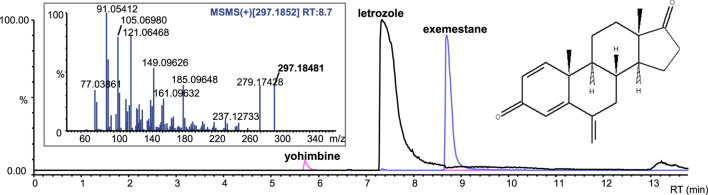
Extracted-ion chromatograms (ESI+) of the sample with letrozole and undeclared exemestane and yohimbine traces; product ion spectrum of exemestane.

Letrozole-containing products on the market include Femara, Aromek, Clarzole, Etruzil, Lametta, and Letrozole. During this study, only two samples with declared letrozole were tested, and both contained the declared API. An example of the letrozole spectrum is shown in Figure 4. However, one letrozole sample contained undeclared exemestane and yohimbine traces (Figure 5). In addition, we identified letrozole as an undeclared API in three samples that were declared to contain anastrozole. One sample contained only letrozole, another contained letrozole with stanozolol, and the third contained letrozole with ostarine (Table 2).

#### 3.5.2 Anastrozole

Anastrozole-containing products on the market include Arimidex, Anastrozol, Anastrozole, Atrozol, and Egistrozol. Seven samples with declared anastrozole were tested; four contained the declared API, two contained only the declared API, the third with the declared API contained undeclared tamoxifen, oxandrolone, ligandrol, and cardarine traces, while the fourth product contained tamoxifen. In contrast, three samples were incorrectly labeled, and the declared API was absent. These samples contained letrozole instead of anastrozole. One sample contained letrozole only, the second contained letrozole and ostarine, and the third contained stanozolol. None of the products tested in this study contained anastrozole as an undeclared active substance (Table 2).

An example of anastrozole spectrum is presented in Figure 4C. Although letrozole and anastrozole share structural similarity and they eluate at the same retention time, their ionization ability in ESI source is completely different. Letrozole ionizes in both positive and negative ionization modes, while anastrozole undergoes only positive ionization.

#### 3.5.3 Exemestane

Exemestane-containing products on the market include Aromasin, Etadron, Glandex, Symex, and Exemestane. Seven samples with declared exemestane content were tested, all of which contained the declared API. However, we found exemestane as an undeclared API in two samples, one with declared chlorodehydromethyltestosterone and undeclared oxandrolone, and the other with declared letrozole and undeclared yohimbine traces (Figure 5; Table 2).

### 3.6 Identification of SERMs and anti-estrogens

All results are summarized in Table 2.

#### 3.6.1 Tamoxifen

Tamoxifen-containing products on the market include Tamoxifen, Zymoplex, and Nolvadex. Seven samples with declared tamoxifen were tested; five contained the declared API, while two were incorrectly labeled, and the declared API was absent. One of these false samples contained ethylenediaminetetraacetic acid disodium salt dihydrate (EDTA-Na), whereas the other sample contained no API. Furthermore, we found tamoxifen as undeclared API in seven samples, together with chlorodehydro-methyltestosterone, stanozolol, anastrozole, oxandrolone, ligandrol, or cardarine (Table 2).

**FIGURE 6:**
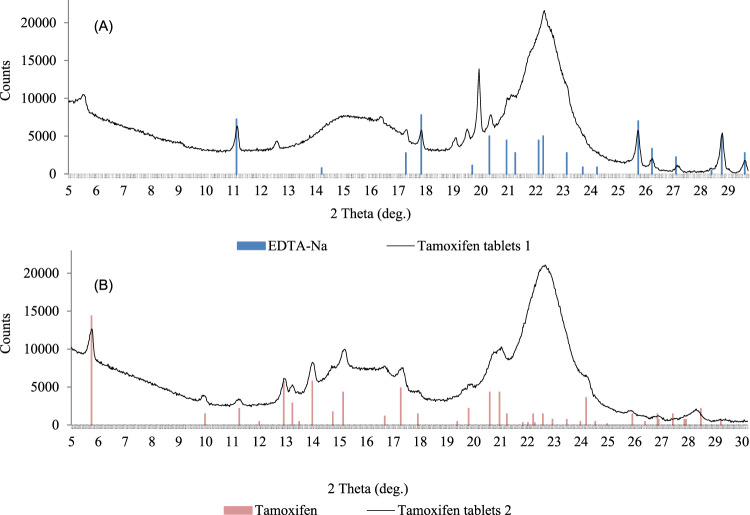
X-Ray diffractograms of tamoxifen samples; tablets without declared tamoxifen but with EDTA-Na **(A)**; tablets with declared tamoxifen citrate **(B)**.

Two sample X-ray diffractograms of the tamoxifen samples are presented in Figure 6.

In sample no. 1 with declared tamoxifen, tamoxifen presence was not confirmed by LC-QTOF-MS/MS or XRPD; instead, another crystalline component, the EDTA-Na salt, was identified (Figure 6A). In sample no. 2, the diffraction measurement results allowed unequivocal tamoxifen identification in the form of citrate (Figure 6B).

#### 3.6.2 Raloxifene

No samples with declared raloxifene were seized. However, in three samples declared as containing AAS or BPC-157, in addition to the declared API, contamination with undeclared raloxifene was present, together with chlorodehydromethyltestosterone, ibutamoren, and SR9009 (Table 2).

#### 3.6.3 Clomiphene

Clomiphene-containing products on the market include Clostilbegyt, Clomiphene Citrate 50, Clomid-50, and Terpafen-50. Fourteen samples with declared clomiphene were tested. Half of the samples contained the declared API, while half were incorrectly labeled, and the declared API was absent. In most of these (n = 4) fake samples, we identified no API, whereas another contained methyltestosterone and stanozolol, the second contained tadalafil and sildenafil, and the third contained methandienone and sildenafil. We identified clomiphene as an undeclared API in five samples, together with tadalafil, sildenafil, dapoxetine, and cardarine (Table 2).

### 3.7 Other PCT substances identification

All results are summarized in Table 2.

#### 3.7.1 Cabergoline

**FIGURE 7:**
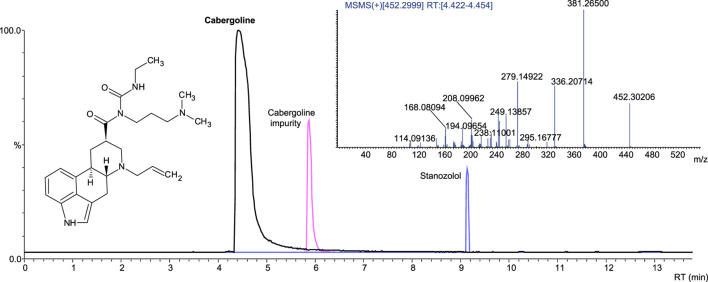
Extracted-ion chromatogram (ESI+) of the sample with cabergoline and stanozolol; product ion spectrum of cabergoline.

Cabergoline-containing medicinal products on the market include Dostinex and Cabaser. Six samples with declared cabergoline were tested; four contained the declared API, while two samples were incorrectly labeled, and the declared API was absent. One of these fake samples contained no API, whereas sildenafil contamination was identified in another. Stanozolol was detected in one sample with declared and identified cabergoline (Figure 7). We did not find cabergoline as an undeclared API in any of the samples (Table 2).

#### 3.7.2 Mesterolone

Mesterolone-containing products on the market include Proviron (at different doses). Four samples with declared mesterolone were tested; two contained the declared API, while two were incorrectly labeled, and the declared API was absent. We identified stanozolol and sildenafil in one of the fake samples and no API in the other. We did not find mesterolone to be an undeclared API in any of the samples (Table 2).

#### 3.7.3 hCG

hCG-containing products on the market include Pregnyl, Vir-Provigil, Ovigil, HCG 5000 IU, Gonadonax 5,000, Chorgon Human Chorionic, Puretrig-5000 IU. Sixteen samples with declared hCG were tested; 10 contained the declared API, while 6 were incorrectly labeled, the declared API was absent, and only mannitol was identified. We did not find hCG as an undeclared API in any sample; however, because of the special identification method, not all samples were screened for hCG (Table 2).

### 3.8 Declared and undeclared API(s) in the PCT samples

Approximately 59% of the PCT samples were correctly labeled, whereas 41% were incorrectly labeled according to the presence of the declared active substances.

**FIGURE 8:**
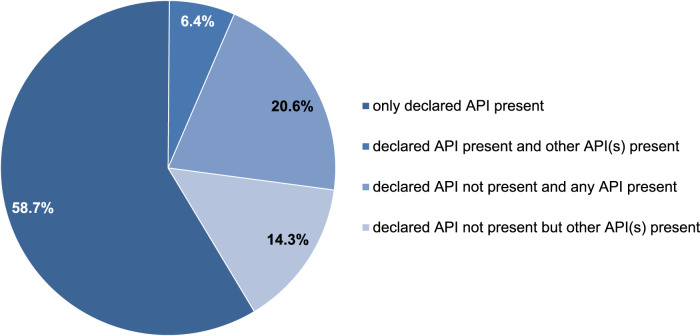
Occurrence of detected APIs within 63 PCT samples.

As described in the previous sections, not all samples were consistent with this declaration. In 65.1% of the PCT samples, the declared API was present, whereas it was absent in 34.9%. Furthermore, in 58.7% of the samples, only the declared API was present, whereas 6.4% contained an additional undeclared API. Conversely, in 20.6% of the samples, neither a declared API nor any API was identified; however, in 14.3% of the samples, other undeclared APIs were identified (Figure 8).

Nine different substances were declared as PCT medicines in the samples and, in order of occurrence frequency, they were hCG (n = 16), clomiphene (n = 14), tamoxifen (n = 7), exemestane (n = 7), anastrozole (n = 7), cabergoline (n = 6), mesterolone (n = 4), and letrozole (n = 2).

In 20 samples, we identified undeclared API used in PCT. Six of these were declared as containing other PCT drugs (of 63); however, 14 other samples should contain only AAS or other substances according declaration (Table 2). Nevertheless, 14 samples declared as containing only AAS and other substances contained undeclared PCT medicines, including tamoxifen (n = 5), clomiphene (n = 5), raloxifene (n = 3), and exemestane (n = 1) (Table 2).

**FIGURE 9:**
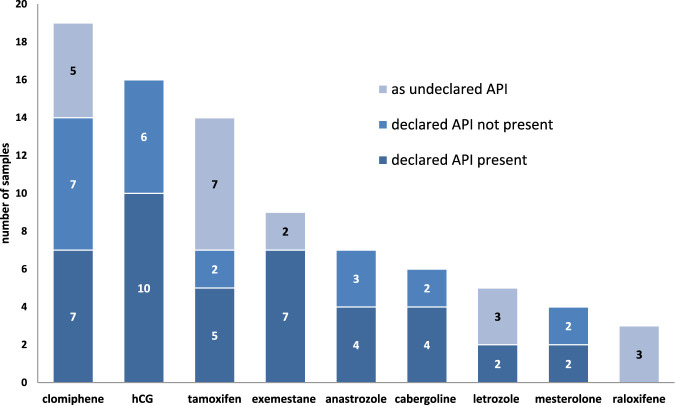
Occurrence of the APIs for PCT in the samples.

A summary of the declared and undeclared APIs in PCT samples is shown in Figure 9.

## 4 Discussion

Between January 2020 and the end of August 2024, 601 PED samples seized by the police and prosecutor’s office from the illegal market were submitted for testing at the Polish OMCL. The fewer samples in 2021 were mainly due to the COVID-19 pandemic in 2020, as samples that reached the laboratory were usually seized much earlier. In contrast, the number of samples received in 2024 was lower; however, this was because the samples were counted only until the end of August. New samples are continually tested in the laboratory, and we typically receive more samples in the second half of the year than in the first half. More than a hundred samples were seized and sent to the Polish OMCL, where they are waiting for analysis.

It is not surprising that AAS products constituted the largest group in each year between 2020 and 2024 (average: 68.4%); however, the number decreased from 80.8% in 2020 to 58.2% in 2023. The year 2024 can only be assessed after completion. Nevertheless, a significant number of AAS products are expected to be analyzed in 2024 because illicit sites that distribute huge quantities of anabolic agents (located in Poland) have recently been tracked down by the police (Centralne Biuro Sledcze Policji, 2023; Centralne Biuro Sledcze Policji, 2024). AAS were also the most dominant group among all analyzed products used as PEDs in other studies (Begley et al., 2017; Deconinck et al., 2021; Magnolini et al., 2022).

Conversely, the number of other PED samples increased (mean 21.1%) from 7.5% in 2020 to 32.9% in 2023. This could be due to a significant increase in interest in SARMs (Deconinck et al., 2021; Gaudiano et al., 2024; Holubeck et al., 2024) or peptide hormones and their releasing factors, growth factor modulators, and metabolic modulators. SARMs have become particularly popular recently among PED users because they are not “steroids” and, therefore, have less stigma attached to them, have lower risks of needle sticks and blood-borne diseases, and appear more legal (Chakrabarty et al., 2021).

Since AAS use is still very popular, the users self-administer post-cycle drugs after an AAS cycle to mitigate the side effects. Self-administration of PCT medicines was reported by 73% of men discontinuing AAS in Scotland between 2015 and 2022 (Grant et al., 2023a) and by 80% of 100 male athletes in the Netherlands between 2015 and 2018 (Smit et al., 2021). However, in an online survey conducted between December 2021 and February 2023, 56.5% of the 470 respondents recruited through advertisements on websites related to AAS use reported PCT use when stopping AAS (Grant et al., 2023b).

In our study, PCT medications constituted 6.5% of samples in 2021, 14.5% in 2022, and 8.5% in 2024 (average, 10.5%). There was a stagnant interest in PCT drugs, as recently demonstrated by other researchers (Holubeck et al., 2024). However, they will not disappear from the market when AAS are still used.

Of the 63 samples, SERMs were declared in 21 (33.3%), AIs in 16 (25.4%), and other PCT substances in 26 (41.3%). Nine different substances were declared or identified as undeclared PCT drugs in the samples and, in order of occurrence frequency, they were clomiphene, hCG, tamoxifen, exemestane, anastrozole, cabergoline, letrozole, mesterolone, and raloxifene (Figure 9). The compounds most frequently encountered in our study were consistent with those described by other researchers. Grant et al. demonstrated that the most popular drugs used for PCT were clomiphene (77%), tamoxifen (75%), and hCG (74%) (Grant et al., 2023a). Notably, hCG was also used during AAS cycle and as a PCT drug together with tamoxifen and clomiphene in 47%, while anastrozole was self-reported by only 4% of respondents. However, according to results of a web-based survey between August 2019 and April 2020 within 2,385 AAS users (Bonnecaze et al., 2020), the main PED used during AAS cycle were anastrozole (48%), tamoxifen (31%), exemestane (30%), and hCG (29%), whereas tamoxifen (40%), clomiphene (32%), hCG (26%), anastrozole (16%), exemestane (9%), letrozole (3%), and cabergoline/bromocriptine (1.6%) were used as PCT; however, 33% of them reported no PCT medicine use. In another study (Smit et al., 2021), tamoxifen (70%), clomiphene citrate (54%), and hCG (55%) were mostly used as PCT drugs. Other researchers analyzed 1792 posts published between 2013 and 2019 on the bodybuilding forum among AAS users, and 845 posts concerned SERMs while 571 concerned AIs (Rochoy et al., 2022).

All the analyzed PCT samples looked like medicines, which may give users confidence in the product quality despite coming from the illegal market. Some evidential product packaging contained anti-counterfeit markings and hints on how to verify product authenticity on the manufacturer’s website. Notably, this is a common “anti-counterfeiting” procedure for manufacturers who illegally produce medicinal products. However, data on the packaging of these products often contain inaccurate information on their composition, origin, performance, and use, which might mislead consumers.

Only over half of the samples were consistent with the declaration. The discrepancies between the label and content can be divided into whether the product contained a similar PED, a completely different API, an additional API, or contained no API. Undeclared pharmacologically active substances in illegal and falsified products can cause extreme harm to public health and pose a serious threat to human life. It is not surprising that there are counterfeits that do not contain API, especially since pharmaceutical API, such as hCG, is expensive compared to other doping agents such as AAS.

Although only three anti-estrogenic substances banned by WADA were identified in this study, clomiphene, tamoxifen, and raloxifene, it should be remembered that other banned substances from the S4.2 group, such as bazedoxifene, cyclofenil, fulvestrant, ospemifene, or toremifene, may also be present in illegal samples purchased by athletes (Okano et al., 2022). Bazedoxifene, ospemifene, and toremifene are newer-generation SERMs that are expected to soon become more popular in the illegal market. Bazedoxifene and ospemifene have been included in the WADA Prohibited List since January 2020. According to blogs, toremifene under the Fareston brand name is even preferred as a modern and safer SERM than tamoxifen and clomiphene; however, it is not yet commonly sold in the black market. In contrast, cyclofenil (Fertodur) is not as commonly used as clomiphene and tamoxifen, which have dominated this drug category for many decades. The SERD fulvestrant is also not yet widely available in the black market (Llewellyn, 2017). This may be due to the fact that it is very expensive, administered as an injection, and must be stored in a refrigerator. Therefore, it is important to note that falsifications without an API can be expected.

Similar to the anti-estrogenic group, only three AIs banned by WADA were identified in this study: letrozole, anastrozole, and exemestane. Notably, other banned substances from the S4.1 group, such as aminoglutethimide, arimistane, formestane, or testolactone, may also be present in illegal samples purchased by athletes. However, letrozole, anastrozole, and exemestane are newer-generation AIs that dominate the drug category. Aminoglutethimide and testolactone (Teslac) were the first generation of non-selective irreversible AIs; however, they are no longer commonly used in clinical medicine. Formestane is a newer AI used as an injection solution and is not widely available in the athletic community (Llewellyn, 2017).

Substances from the newer generations have started appearing among athletes. Within the class S4 of “hormone and metabolic modulators” of the WADA Prohibited List, 39% of the adverse analytical findings (AAF) reported in 2020 resulted from SERMs (tamoxifen and clomiphene) and another 29% from AIs (letrozole, anastrozole, exemestane, and adrostatrienedione) (World Anti-Doping Agency, 2022). In 2021, the numbers of AAF were 33% from SERMs (tamoxifen and clomiphene) and 15% from AIs (anastrozole, letrozole, adrostatrienedione, armistane, and 2-androstenone) (World Anti-Doping Agency, 2024a). Further, the numbers were 35% from anti-estrogenic substances (tamoxifen, clomiphene, fulvestrant, toremifene, and raloxifene) and 20% from AIs (letrozole, anastrozole, exemestane, adrostatrienedione, and armistane) in 2022 (World Anti-Doping Agency, 2024b).

Among the other PCT drugs, bromocriptine, which is similar to the popular cabergoline, an ergot alkaloid derivative used to treat hyperprolactinemia, is less common than cabergoline among AAS users during PCT owing to its many side effects, such as low blood pressure, dizziness, confusion, and nausea.

The effectiveness and purity of PCT drugs are concerns for many users (Grant et al., 2023b). Although many AAS users believe that PEDs are safe for long-term use, the vast majority would stop using AAS if they experience a serious health problem (Bonnecaze et al., 2020). In another study was reported that respondents believed that confirming substance legality would encourage safer practices and reduce potentially dangerous side effects (Piatkowski et al., 2023).

Polypharmacy associated with AAS and PCT drugs may have serious harmful effects on those engaged in such practices, which constitutes a serious public health concern (Sagoe et al., 2015). Moreover, illegal and falsified products used in PCT may interact with other medications or affect preexisting disease development or treatment.

Although these medications are legally available only with the prescription of a doctor, they can be bought without a prescription on the Internet. In another study researchers analyzed posts on a bodybuilding forum concerning SERMs and AIs, revealing that a minority of users were able to obtain PCT medication through a pharmacy (5% clomiphene, 5% tamoxifen, 6% anastrozole, 8% exemestane, and 22% letrozole) (Rochoy et al., 2022). Only 5% of AAS users reported consulting physicians. However, many of them (25% of SERM and 33% of AI users) reported having a blood test to monitor levels of gonadotropins, estradiol, and total, free, and bioavailable testosterone and to adjust AI doses during AAS treatment. It should be emphasized that in another survey (Grant et al., 2023b), most respondents believed that PCT should be prescribed under the supervision of a physician. However, no recommended treatment for AAS cessation currently exists. PCT use after discontinuing AAS treatment reduced the desire to reuse AAS, withdrawal symptoms, and suicidal thoughts by 60%, 60%, and 50%, respectively (Grant et al., 2023b). However, SERM, AI, or prolactin inhibitor use during PCT remains an unproven practice (Bond et al., 2022). Some researchers were unable to demonstrate any beneficial effect of PCT medications; therefore, the use of these agents should be discouraged (Smit et al., 2021).

Owing to the high prevalence of illicit steroid use, the self-administration of unapproved PCT using illegal and falsified medicines is dangerous and can be considered a potential threat to consumer health. Pharmaceutical and drug-related crimes are problems that the modern world struggles with. The increasing scale of this phenomenon, dynamic changes in the market for falsified and illegally traded products, and threats to the health and life of people receiving such products imply the urgency of systematic monitoring of both legal and illegal chains of medicinal products.

We have shown that the illicit drugs used in PCT are of poor quality; they may be substituted, contain no active ingredients, or be adulterated. Our results indicate that in view of the high prevalence of illicit AAS use, the self-administration of unapproved PCT using illegal and falsified medicines is dangerous and can be considered a potential threat to consumer health.

### 4.1 Limitations

This study had some limitations. Because only qualitative analysis was performed, the exact doses of illicit PCT drugs were unknown. Therefore, even samples where the labeled API was confirmed may not be consistent with the declaration because of variations in API concentrations, which may have health implications for consumers.

Not only APIs but also declared excipients were analyzed, mainly by XRPD; however, without the full composition of the analyzed products (known only as legal medicinal products), confirming compliance with the declaration was not possible.

## Data Availability

The raw data supporting the conclusions of this article will be made available by the authors, without undue reservation.

## Abbreviations

AAF, adverse analytical finding; AAS, anabolic-androgenic steroid; AI, aromatase inhibitor; API, active pharmaceutical ingredient; EDTA-Na, ethylenediaminetetraacetic acid sodium salt; ER, estrogen receptor; DTT, dithiothreitol; hCG, human chorionic gonadotropin; LC-MS, liquid chromatography-mass spectrometry; LC-QTOF-MS/MS, high-resolution hybrid quadrupole-TOF mass spectrometry coupled with high performance liquid chromatography; MS, mass spectrometry; OMCL, Official Medicines Control Laboratory; PCT, post-cycle therapy; PDE-5i, phosphodiesterase-5 inhibitor; PED, performance-enhancing drug; PTFE, polytetrafluoroethylene; Q, quadrupole; SARM, selective androgen receptor modulator; SERM, selective estrogen receptor modulator; SERD, selective estrogen receptor degrader; TOF, time-of-flight; WADA, World Anti-Doping Agency; XRPD, X-ray powder diffraction.
